# Assessment of the efficacy of hemodialysis on uric acid clearance in a sub-Saharan African population at the end stage kidney disease

**DOI:** 10.1186/s12882-020-02037-8

**Published:** 2020-08-31

**Authors:** Marie Doualla, Jan René Nkeck, Marie Patrice Halle, Félicité Kamdem, Aude Ingrid Agouak, Mickael Essouma, Yonathan Batchama Lobe, Gloria Ashuntantang

**Affiliations:** 1 Faculty of Medicine and Biomedical Sciences, University of Yaoundé I, Yaoundé, Cameroon; 2 Faculty of Medicine and Pharmaceutical Sciences, University of Douala, Douala, Cameroon; 3 Douala General Hospital, Douala, Cameroon; 4 Faculty of Health Sciences, University of Buea, Buea, Cameroon

**Keywords:** Chronic Kidney Disease, hyperuricemia, hemodialysis

## Abstract

**Background:**

Uricemia dramatically rises with the stage of chronic kidney disease (CKD) and correlates with its mortality. Hemodialysis (HD) being the most used treatment at the end stage in sub-Saharan Africa, we sought to evaluate its efficacy on the clearance of uric acid (UAc) when used alone and twice per week.

**Methods:**

A cross-sectional study of all consenting patients with CKD stage 5 recruited at random during HD sessions in a reference Centre in Cameroon from January to April 2017. We collected socio-demographic data, relevant clinical information, HD related variables, and measured serum uric acid (SUA) levels before and after the dialysis to assess the uric acid clearance. A clearance between 65 and 80% and above 80% was considered as low and good efficacy of HD respectively. Statistical analysis was performed using SPSS version 21.0. Factors associated with HD efficacy were assessed using Fisher’s exact test and are presented with their odds ratios (OR) and 95% confidence levels.

**Results:**

One hundred four patients (53 females) were included. The mean age was 49.9 ± 13.3 years. Hypertension (25%) and chronic glomerulonephritis (16%) were the main suspected etiologies of CKD. The median time on renal replacement therapy by HD was 3 years [1; 6]. The prevalence of hyperuricemia was 81.9%. The means of SUA levels were 78.8 ± 13.8 mg/L and 26.4 ± 6.6 mg/L respectively before and after dialysis. Mean SUA clearance was 66% ± 10%. The efficacy of HD on UAc was moderate in 92 (63.9%) and good in 2 (1.4%) patients. Excess weight (OR 0.4 [0.2; 0.9]) and Kt/Vurea < 1.2 (OR 0.1 [0.04; 0.2]) significantly reduces the efficacy of HD.

**Conclusion:**

HD used alone for 2 sessions per week has a moderate efficacy on uric acid clearance in CKD. Therefore, we should improve the Kt/V (> 1.2), and combine HD to uric acid lowering drugs and diet modifications to increase its efficacy.

## Background

Chronic Kidney Disease (CKD) is a global health problem that affects more than 200 million people around the world [1]. Africa has the highest prevalence of CKD and about 10–23% of adults are affected in the sub-Saharan region [2–4]. In Cameroon, CKD is estimated to affect 13.2% of adults and the proportion of people with ESKD is certainly high but the exact figures remain unknown [5]. At end-stage kidney disease (ESKD), maintenance hemodialysis (HD) is the most used course of treatment. In addition to renal replacement therapy, physicians need to consider various types of medications in order to tackle the numerous complications associated with it and reduce the burden of disease. The prevalence of hyperuricemia dramatically increases with the stage of CKD and affects approximately 70% of patients with CKD stage 4 or 5 [6, 7]. The association of ESKD and hyperuricemia may amplify the inflammatory processes, oxidative stress and endothelial dysfunction which are the basis for the development and worsening of cardiovascular damages, the first cause of death in ESKD patients [6, 8–11]. Increased serum uric acid levels (SUA) may correlate and predict cardiovascular mortality in HD patients [7, 12]. Thus, reducing SUA appears to be crucial in the treatment of ESKD and may significantly impact patients’ survival [7, 13].

Of the various strategies in place for the management of hyperuricemia during ESKD, HD alone has been shown to be the most effective [14, 15]. However, there is still a gap and some controversies regarding countries being out of line with the KDOQI’s recommendations according to the number of HD sessions per week [16, 17]. In this situation, few data are available to support the use of HD alone or with the addition of a uric acid-lowering therapy (UALT). The state of care for ESKD patients in sub-Saharan Africa is of concern. Several factors including the low socioeconomic status, poor access to quality care and a low nephrologist to patient ratio contribute to the high mortality of ESKD patients, most of whom only have access to two HD sessions in a week [18, 19]. Efficient strategies have to be developed to reduce the potential contribution of hyperuricemia to the burden of ESKD in this population. Hence, we sought to estimate the efficacy of HD alone for uric acid clearance in patients with ESKD and evaluate the need to add a UALT. This study will add to the already available data on the management of hyperuricemia in ESKD patients in a low resource setting.

## Methods

The methods section was presented according to the STROBE recommendations for observational studies [20].

### Study design and Setting

We carried out a prospective cross-sectional study at the Hemodialysis Centre of the Douala General Hospital (DGH). It is a tertiary and referral hospital in the urban city of Douala, equipped with 18 dialyzers branded Fresenius® 4008S generator (Fresenius Medical Care, Hamburg, Germany), with a polysulfone synthetic dialysis membrane and bicarbonate dialysates. It is the largest dialysis center in the country, caring for 225 patients by the end of the year 2016. Study participants were recruited from the 1st January to 31st April 2017 (4 months). Eighty percent of the patients received two dialysis sessions per week.

### Participants

We included all consenting adults (≥21 years), at the end-stage kidney disease according to the KDIGO 2012 [21], who were undergoing regular hemodialysis for 3 months at the DGH. We excluded patients who were on UALT, who received less than 4 h of dialysis and those who had emergency dialysis. All the participants were approached during a random HD session.

### Sample size estimation

The sample size was estimated by convenience in order to recruit at least 60% of the patients which were already followed in the hemodialysis unit by the end of the year 2016, i.e. 135 participants.

### Data measurement

We used a data extraction form to report information about the gender, age, underlying nephropathy, average time on renal replacement therapy, comorbidities, residual diuresis (< 100 ml/24 or ≥ 100 ml/24 h) of participants; and current hemodialysis session parameters such as vascular access, duration of the session, time interval between the current and previous dialysis session, body mass index (BMI), blood pressure at the beginning and at the end of dialysis, dialysis adequacy, blood efflux (< 300 ml/min or > 300 ml/min), and serum uric acid level which were assessed on a peripheral blood sample collected 5 min before and after dialysis. Uricemia was determined by the enzymatic and colorimetric method called uricase [22].

### Operational terms

Hyperuricemia was considered for uric acid levels above 70 mg/L for men and 60 mg/L for women [23]. Excess weight was defined as a BMI above 25 Kg/m^2^. The size of the dialysis membrane was estimated according to the total body surface of the patient. Membranes sizes FX5 were used for a body surface of approximately 1.0 m^2^; whereas FX8 and FX10 were used for body surfaces respectively between 1.4and 1.8 m^2^ and above 1.8 m^2^. Dialysis adequacy was calculated from the formula Kt/Vurea; K being the clearance of urea given by the dialyzer, t the duration of the dialysis session (4 h) and V the distribution volume of urea [24]. Uric acid clearance (UAc) was estimated using the formula: UAc = 100 x (SUA levels before HD –SUA levels after HD) / SUA levels before HD] [14]. We defined two thresholds values for UAc (65 and 80%). A HD session was considered to be of low efficacy if below 65% of reduction, moderate efficacy between 65 and 80% and a good efficacy above 80% of UAc. As there is currently no recommended threshold for uric acid clearance, these thresholds have been defined by an expert consensus adapting to our population the KDIGO 2012 recommendations for urea, set at 65% for the general population performing 3 sessions of hemodialysis per week. The threshold of good efficacy have been set at 80%, above the urea’s clearance objective recommended by the KDIGO 2012. According to the study objective, a higher threshold for good efficacy was used for our study population who have a lower number of hemodialysis sessions per week, and would therefore be expected to have a higher prevalence of pre-dialysis hyperuricemia than the general population.

### Statistical analysis

All the data collected were analyzed using the software SPSS version 21.0. Quantitative variables were expressed in terms of mean and standard deviation (SD) or median with the interquartile range [Q25; Q75], while qualitative variables were expressed as counts and proportions. The comparison of means of paired quantitative variables was done using the paired Student’s T-test. Factors modifying UAc were determined using Fisher’s exact test. The odds ratio (OR) and its 95% confidence interval were used to quantify the degree of association. The threshold of significance was set at 0.05 for all the statistical tests used.

## Results

### Baseline characteristics of the study population

Overall 144 patients (63.2% males) were involved in the study. The mean age of participants was 49.2 ± 14.1 years. Underlying nephropathies included hypertensive nephropathy (25%), chronic glomerulonephritis (16%), diabetic nephropathy (15.3%), and were unknown in 27 patients (18.8%). At the time of the study, 128 (88.9%) patients were reported to have hypertension, 47 (32.6%) had excess weight, and 33 (22.9%) had diabetes. 124 (86.1%) patients had an arterio-venous fistula (86.1%), and 105 (72.9%) had a kidney size about FX10.

Hyperuricemia was found in 118 patients (81.9%) and 16 (11.1%) had a documented gout crisis in their past history since the initiation of hemodialysis. Ninety-one (63.2%) patients reported less than 100 mL urine output in 24 h. The baseline characteristics of the population are presented in Table 1.

**Table 1:** Baseline characteristics of the study population

Variables		Overall	Males	Females
N (%)		144 (100)	91 (63.2)	53 (36.8)
Mean age, year, (SD)		49.2 (14.1)	50 (14)	48 (15)
Age, min-max, year		15–74	22–74	15–74
Underlying nephropathy, n (%)	Hypertensive nephropathy	36 (25)	28 (19.4)	8 (5.6)
	Unknown	27 (18.8)	13 (9)	14 (9.7)
	Diabetic nephropathy	22 (15.3)	18 (12.5)	4 (2.8.)
	Chronic glomerulonephritis	23 (16)	16 (11.1)	7 (4.9)
	Chronic interstitial nephropathy	8 (5.6)	8 (5.6)	0 (0)
	Gout nephropathy	7 (4.9)	0 (0)	7 (4.9)
	Polycystic kidneys	7 (4.9)	1 (0.7)	6 (4.2)
	Hypertension and diabetes	6 (4.2)	4 (2.8.)	2 (1.4)
	HIV associated nephropathy	6 (4.2)	3 (2.1)	3 (2.1)
	Hepatitis C nephropathy	1 (0.7)	1 (0.7)	0 (0)
	Focal and segmental hyalinosis	1 (0.7)	0 (0)	1 (0.7)
Comorbidities, n (%)	Hypertension	128 (88.9)	84 (58.3)	44 (30.6)
	Hyperuricemia	118 (81.9)	71 (40.9)	47 (32.6)
	Excess weight	47 (32.6%)	30 (20.8)	17 (11.8)
	Diabetes	33 (22.9)	23 (16)	10 (6.9)
	Cardiopathies	24 (16.7)	17 (11.8)	7 (4.9)
	Past history of cerebral stroke	12 (8.4)	9 (6.3)	3 (2.1)
	Hepatitis C	18 (12.5)	12 (8.3)	6 (4.2)
	HIV	7 (4.9)	3 (2.1)	4 (2.8)
	Hepatitis B	3 (2.1)	3 (2.1)	0 (0)
	Past history of gout	16 (11.1)	14 (9.7)	2 (1.4)
	Tobacco consumption	4 (2.8)	4 (2.8)	0 (0)
Mean BMI, Kg/m^2^ (SD)		23.8 (3.6)	23.6 (2.7)	24.1 (4.9)
Residual diuresis, n (%)	< 100 mL/24 h	91 (63.2)	61 (42.4)	30 (20.8)
	≥100 mL/24 h	53 (36.8)	30 (20.8)	23 (16)
Median time on HD replacement [Q25-Q75], year		3 [1; 6]	3 [1; 6]	2 [1; 5]
Interval between dialytic sessions, n (%)	< 3 days	24 (16.7)	15 (10.4%)	9 (6.3%)
	≥3 days	120 (83.3)	76 (52.8%)	44 (30.6%)
Vascular access, n (%)	Fistula	124 (86.1)	79 (54.9)	45 (31.3)
	Catheter	20 (13.9)	12 (8.3)	8 (5.6)
Size of the dialysis membrane, n (%)	FX10	105 (72.9)	73 (50.7)	32 (22.2)
	FX8	37 (25.7)	17 (11.8)	20 (13.9)
	FX5	2 (1.4)	1 (0.7)	1 (0.7)
Blood efflux, n (%),	< 300 ml/min	75 (52.1)	43 (29.9)	32 (22.2)
	≥300 ml/min	69 (47.9)	48 (33.3)	21 (14.6)
Mean Kt/VUrea (SD)		1.3 (0.2)	1.2 (0.2)	1.5 (0.2)

### Hemodialysis parameters

The median duration on maintenance HD was 3 [1; 6] years. The duration between dialysis sessions was at least 3 days for 120 (83.3%) participants. The mean Kt/Vurea was 1.3 ± 0.2 and could only be calculated for 34 participants (Table 1). The mean systolic blood pressure was significantly increased after dialysis (*p* = 0.02), but the mean of the diastolic blood pressure remained unchanged (*p* = 0.10) (Table 2).

**Table 2:** Evolution of blood pressure and uricemia with hemodialysis

Variables	Before dialysis	After dialysis	*p*-value
Mean Systolic blood pressure, mmHg (SD)	153 (25)	157 (30)	**0.02**
Mean Diastolic blood pressure, mmHg (SD)	82 (18)	85 (19)	0.1
Mean Uricemia, mg/L (SD)	78.8 (13.8)	26.4 (6.6)	**< 0.001**

### Uric acid clearance and efficacy of HD

**Fig. 1:**
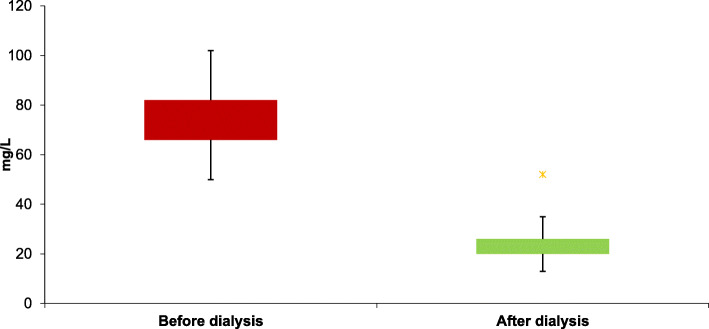
Serum uric acid levels before and after the hemodialysis

The mean SUA level before hemodialysis was 78.8 ± 13.8 mg/L and the mean SUA level after hemodialysis was 26.4 ± 6.6 mg/L (Table 2). HD significantly reduces SUA level (*p* < 0.001) (Fig. 1). The mean UAc after HD was 66% (±10). The efficacy of HD on UAc was moderate in 92 (63.9%) patients and good in 2 (1.4%) patients. A Kt/Vurea less than 1.2 (*p* = 0.01, OR 0.1 [0.04; 0.2]) and an excess weight (*p* = 0.03, OR 0.4 [0.2; 0.9]) were found to be significantly associated to HD efficacy (Table 3). The other patient and HD related variables were not statistically associated to better UAc (Table 3).

**Table 3:** Associated factors of uric acid clearance on hemodialysis in ESKD

Variables	Uric acid Clearance		***p***-value	OR [95% CI]
	≥ 65%	< 65%		
**N (%)**	94 (100)	50 (100)		
**Age, n (%)**				
< 50 years	47 (50)	26 (52)	0.8	–
≥ 50 years	47 (50)	24 (48)		
**Sex, n (%)**				
Men	56 (59.6)	35 (70)	0.2	–
Women	38 (40.4)	15 (30)		
**Comorbidities**				
**Excess weight, n (%)**				
Yes	25 (26.6)	22 (44)	**0.03**	**0.4 [0.2; 0.9]**
No	69 (73.4)	28 (56)		
**Hypertension, n (%)**				
Yes	84 (89.4)	44 (88)	0.8	–
No	10 (10.6)	6 (12)		
**Diabetes, n (%)**				
Yes	20 (21.3)	13 (26)	0.5	–
No	74 (78.7)	37 (74)		
**Gout, n (%)**				
Yes	8 (8.5)	8 (16)	0.1	–
No	86 (91.5)	42 (84)		
**Underlying nephropathy**				
**Hypertensive nephropathy, n (%)**				
Yes	57 (60.6)	31 (62)	0.8	–
No	37 (39.4)	19 (38)		
**Diabetic nephropathy, n (%)**				
Yes	80 (85.1)	39 (78)	0.2	–
No	14 (14.9)	11 (22)		
**Chronic glomerulonephritis, n (%)**				
Yes	80 (85.1)	41 (82)	0.6	–
No	14 (14.9)	9 (18)		
**Hemodialysis related variables**				
**Blood efflux, n (%)**				
< 300 ml/min	49 (52.1)	26 (52)	0.9	–
> 300 ml/min	45 (47.9)	24 (48)		
**Interval between dialytic sessions, n (%)**				
< 3 days	16 (17)	8 (16)	0.8	–
≥ 3 days	78 (83)	42 (84)		
**Size of the dialysis membrane, n (%)**				
FX 10	67 (71.3)	38 (76)	0.5	–
FX 8 or FX 5	27 (28.7)	12 (24)		
**Kt/Vurea**				
< 1.2	22 (23.4)	38 (76)	**0.01**	**0.1 [0.04; 0.2]**
≥ 1.2	72 (76.6)	12 (24)		

## Discussion

Proven to be associated with a poor outcome, hyperuricemia has to be treated in patients with ESKD. The aim of this study was to determine the efficacy of hemodialysis on uric acid clearance when done twice a week, in order to evaluate the need for adjunctive UALT. We found an average clearance above 65%, indicating a moderate efficacy. An increase in certain parameters such as Kt/Vurea may improve this efficacy without the need for additional treatment.

Hyperuricemia is frequent and may have vascular consequences in ESKD. We found an elevated prevalence of hyperuricemia in ESKD which is in accordance with the available literature, [6, 7, 14]. However, the cumulative incidence of gout attack since the initiation of dialysis was low (11.1%); similar to the findings previously reported by Eleftheriadis et al. HD significantly decreased the number of gout attacks [25], however, gout is not the only consequence of hyperuricemia in ESKD. Other pathological effects of hyperuricemia apart from gout result in inflammation, oxidative stress and endothelial dysfunction [7–11], with major effects on lipids, causing vascular damages, and leading to increased rates of cardiovascular complications like cerebral stroke and cardiopathies, which were reported in our sample [10]. An increase in SUA levels has been reported to be a predictive factor of cardiovascular diseases in patients undergoing HD, which is the major cause of mortality in ESKD [7].

We, therefore, sought to evaluate the efficacy of HD alone on uric acid clearance. We observe that HD alone significantly reduces uricemia (*p* < 0.001), but, this reduction was moderate (mean UAc of 66 ± 10%). A few participants (1.4%) had more than 80% clearance, which corresponds to those undergoing 3 sessions of HD per week. This result is similar to those of Alaraj et al. in Saudi Arabia [26] who found a reduction of 66.14% (±18.8) with dialysis. However, it differs from those of Soriano et al. in Spain, who found a larger proportion (56.7%) of patients with more than 80% reduction [14]. This difference could be explained by the fact that Soriano et al. worked on a population who had been on maintenance HD for a long time (median 7 years) and received 3 sessions per week, which was not the case in our study, where the median of hemodialysis was 3 [1; 6] years and most participants received 2 sessions of HD per week. Furthermore, they had a greater percentage of patients reporting gout crisis (21.6%) who were certainly on diet modifications to lower uric acid levels and of which 16.4% were on uric acid lowering drugs at the time of the study. In our study, we assessed the efficacy of hemodialysis alone, thus, all the participants on UALT were excluded.

To improve the efficacy of hemodialysis, we assessed the factors associated with good efficacy. We found that the efficacy of HD on SUA level is influenced by dialysis adequacy (Kt/Vurea). A Kt/Vurea < 1.2 significantly reduced the efficacy of HD with an OR of 0.1 [0.01; 0.6]. The dialysis adequacy assessed by Kt/Vurea measures how much urea is removed during dialysis and has been adopted to 1.2 as standard value for dialysis adequacy by the Kidney Disease Outcomes Quality Initiative (KDOQI) [24]. A Kt/V above 1.2 improves the efficacy of HD and could be achieved by an increase in blood flow through the dialyzer to 600 or 800 ml/min and/or an increase the time on dialysis [24]. However, the blood efflux in our study was not associated with HD efficacy on uric acid clearance (*p* > 0.05) and the duration of dialysis was already set at 4 h for all the participants. Soriano et al. found that a Kt/v < 1.3 and blood efflux < 400 mL/min was significantly associated to achieve a reduction above 80% [14]. These results are contrary to ours and is certainly due to the difference in materials (dialyzer, dialysis membrane, dialysates) used for hemodialysis. All other HD-related variables except the size of the dialysis membrane (*p* = 0.5) did not have statistical influence on uric acid clearance. A prolonged interval between dialysis sessions (≥ 3 days), known to be associated with the occurrence of hyperuricemia in literature [27], was not found to have an influence on the efficacy of HD in our study.

Apart from HD related variables, the participant’s characteristics were tested for association with UAc. We found that excess weight significantly reduces uric acid clearance (*p* = 0.03, OR 0.4 [0.2; 0.9]. The weight of participants defines the size of the dialysis membrane and weight influences the volume of distribution and thus the plasma clearance of the dialyzed substances. Age (*p* = 0.8), sex (*p* = 0.2), and comorbidities like hypertension (*p* = 0.8), diabetes (*p* = 0.5) and gout (*p* = 0.1) were not associated to a better clearance. These results are similar to those of Alaraj et al. and Soriano et al. [14, 26]. Moreover, no association was found between underlying nephropathies (hypertensive, diabetic nephropathies, etc.) and the efficacy of hemodialysis.

An average efficacy above 65% is a good value for clinicians in order to ensure the reduction in SUA level and thus the incidence of hyperuricemia associated damages and manifestations. The use of HD in combination with diet modification and UALT could best increase its efficacy to prevent hyperuricemia as reported for allopurinol and febuxostat [15, 28, 29]. Although our findings are important, they should be considered and interpreted in light of some limitations: (1) the small sample size which may be improved in a multicenter study; (2) the long-term outcome regarding the efficacy of hemodialysis on uric acid clearance which is lacking and may be enhanced by multiple measurements of SUA levels, given that SUA may vary with time, and a long term evaluation of the effects of hemodialysis parameters; (3) the lack of strong criteria assessing diet which is a great source of purine and thus has an impact on SUA levels.

## Conclusion

Despite being the main choice for renal replacement therapy in patients with ESKD in low income countries, hemodialysis alone for 2 sessions per week, has a moderate efficacy on uric acid clearance. To increase its efficacy, practitioners could increase the Kt/V (> 1.2) and combine HD to uric acid lowering drugs and diet modifications.

## Data Availability

The datasets used for this study are available from the corresponding author on reasonable request.

## Abbreviations

BMI: Body mass index
CKD: Chronic kidney disease
DGH: Douala general hospital
ESKD: End stage kidney disease
HD: Hemodialysis
SUA: Serum uric acid
UAc: Uric acid clearance
UALT: Uric acid lowering therapy
